# Mechanistic movement models reveal ecological drivers of tick-borne pathogen spread

**DOI:** 10.1098/rsif.2021.0134

**Published:** 2021-08-11

**Authors:** Olivia Tardy, Catherine Bouchard, Eric Chamberland, André Fortin, Patricia Lamirande, Nicholas H. Ogden, Patrick A. Leighton

**Affiliations:** ^1^ Research Group on Epidemiology of Zoonoses and Public Health (GREZOSP), Faculty of Veterinary Medicine, Université de Montréal, 3200 rue Sicotte, Saint-Hyacinthe, Québec, Canada J2S 2M2; ^2^ Groupe Interdisciplinaire de Recherche en Éléments Finis (GIREF), Department of Mathematics and Statistics, Faculty of Science and Engineering, Université Laval, 1045 avenue de la Médecine, Québec, Québec, Canada G1V 0A6; ^3^ Public Health Risk Sciences Division, National Microbiology Laboratory, Public Health Agency of Canada, 3200 rue Sicotte, Saint-Hyacinthe, Québec, Canada J2S 2M2; ^4^ Centre for Public Health Research (CReSP), Université de Montréal and the CIUSSS du Centre-Sud-de-l’Île-de-Montréal, 7101 avenue du Parc, Montréal, Québec, Canada H3N 1X9

**Keywords:** boosted regression tree, *Borrelia burgdorferi*, host movement, *Ixodes scapularis*, reaction–advection–diffusion model, tick-borne disease

## Abstract

Identifying ecological drivers of tick-borne pathogen spread has great value for tick-borne disease management. However, theoretical investigations into the consequences of host movement behaviour on pathogen spread dynamics in heterogeneous landscapes remain limited because spatially explicit epidemiological models that incorporate more realistic mechanisms governing host movement are rare. We built a mechanistic movement model to investigate how the interplay between multiple ecological drivers affects the risk of tick-borne pathogen spread across heterogeneous landscapes. We used the model to generate simulations of tick dispersal by migratory birds and terrestrial hosts across theoretical landscapes varying in resource aggregation, and we performed a sensitivity analysis to explore the impacts of different parameters on the infected tick spread rate, tick infection prevalence and infected tick density. Our findings highlight the importance of host movement and tick population dynamics in explaining the infected tick spread rate into new regions. Tick infection prevalence and infected tick density were driven by predictors related to the infection process and tick population dynamics, respectively. Our results suggest that control strategies aiming to reduce tick burden on tick reproduction hosts and encounter rate between immature ticks and pathogen amplification hosts will be most effective at reducing tick-borne disease risk.

## 1. Introduction

Host movement is a fundamental component for predicting pathogen invasion dynamics [1]. For pathogens that are transmitted by the bite of arthropod vectors, disease spread is favoured by the movement of both infected hosts and vectors [2]. Ticks of public health importance are often generalist parasites, with a large number of host species that differ in their reservoir competence [3], their ability to disperse ticks [4] and their habitat use [5]. Hosts, therefore, play a central role in the distributional dynamics of tick-borne pathogens at different spatial scales [6].

Host movement patterns, influenced by landscape composition and structure, can create strong spatial heterogeneity in contact rates with arthropod vectors [7]. In tick-borne disease systems, results from modelling studies suggest that microhabitat conditions and climate change are not sufficient to explain the variability observed in patterns of tick abundance and that dispersal of ticks depends on how hosts perceive and respond to their ecological and social environments (e.g. [8,9]). Theoretical models and empirical observations of foraging movement behaviour suggest that animals decrease their movement speed and frequently turn in resource-rich areas, while in areas with low resource abundance, animal movement is more directed with greater distances between successive locations (e.g. [10,11]). Previous modelling studies for tick–host–pathogen systems (e.g. [12,13]) modelled host movement using uncorrelated random walks, which can lead to unrealistic animal movements [14] because these models do not account for directional persistence in movement (i.e. tendency to move in the same direction) or directional bias towards a centre of attraction (e.g. den or food site). This calls into question the accuracy of predictions about the spatial distribution of ticks and their pathogens resulting from models that do not consider the directional movement of hosts.

Mechanistic home-range models [15,16] offer a promising approach to modelling the movement of animals in heterogeneous landscapes. These models incorporate random walks in which animal movement is characterized by distributions of step lengths and turning angles, with home-range behaviour described by a correlated random walk incorporating a directional bias towards a centre of attraction. Mechanistic home-range models have been used to analyse a large variety of ecological processes such as avoidance response to habitat edges resulting from anthropogenic disturbances [17], memory [18], territoriality and habitat selection [11]. Although such models offer an elegant approach to describing spatial dynamics of wildlife pathogens in heterogeneous landscapes, they have yet to see a widespread application in the fields of disease ecology and epidemiology.

The bacterium *Borrelia burgdorferi* sensu stricto is responsible for Lyme disease, the most prevalent vector-borne zoonotic disease in North America [19]. This pathogen is transmitted from host to host through the bite of hard-bodied ticks of the *Ixodes* genus [20]. *Ixodes* ticks have four stages of development in their life cycle: egg, larva, nymph and adult. Larval and nymphal ticks are the main vectors of *B. burgdorferi* and are generalists, feeding on a large variety of vertebrate hosts with different body sizes including mammals, birds and reptiles [21]. In contrast, adult female ticks require a blood meal from large-bodied mammals such as deer to mate with an adult male tick and then lay eggs [22]. Given the range expansion of *B. burgdorferi* from northeastern and midwestern regions of the United States since the late 1970s and the associated spread of Lyme disease risk in North America [23], there is an urgent need to identify ecological drivers that influence the ongoing spread of this pathogen into new regions.

In the United States, the emergence of endemic areas for *B. burgdorferi* has been attributed to post-agricultural reforestation and the expansion of deer populations since the mid-twentieth century [19]. Because deer are incompetent reservoir hosts for *B. burgdorferi* [24] and move relatively short distances (maximum home range diameter of 5 km), this host species is especially important for the local dispersal of ticks into new areas and helps maintain tick populations [25]. In North America, bird species are known to play a major role in the spread of both *B. burgdorferi* and *I. scapularis* ticks over long distances [26]. At least 71 species of North American birds can be parasitized by *I. scapularis* ticks and 60% of these species can serve as competent reservoirs for *B. burgdorferi* [27]. Most studies seeking to predict range expansion of the bacterium *B. burgdorferi* and *I. scapularis* ticks in North America have focused on environmental and climatic conditions that allow tick populations to persist, grow or colonize, assuming these conditions contribute to habitat and climatic suitability for ticks (e.g. [28,29]). However, little consideration has been given to how the long-distance movement behaviour of birds and their response to landscape heterogeneity affects the spread of tick-borne pathogens.

In this study, we built a mechanistic movement model to explore how the interplay between host movement, tick and host demographic processes, the spatial distribution of resources and the pathogen infection process influences tick-borne pathogen spread dynamics across spatially heterogeneous landscapes. To do this, we ran a series of simulations of northward invasion of ticks by migratory birds and terrestrial hosts across theoretical landscapes that differed in spatial aggregation (clustering) of resources.

## 2. Material and methods

### 2.1. Model formulation

**Figure 1.**
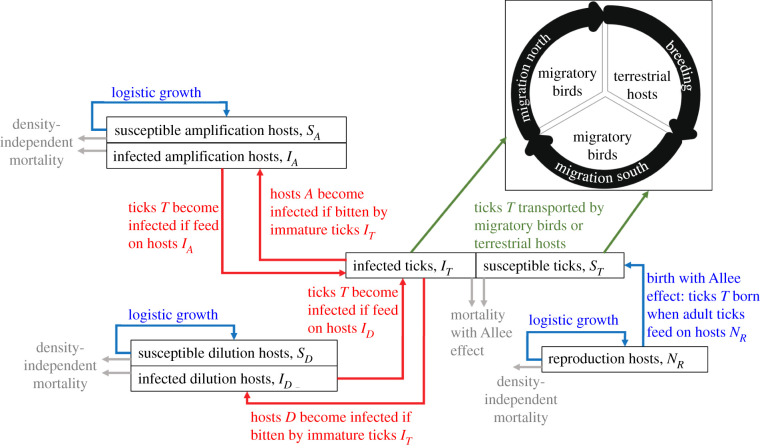
Theoretical framework describing the spread of a tick population and its infection with a pathogen in a system of amplification, dilution and reproduction hosts. Solid red lines indicate the transmission cycle of the pathogen circulating in the tick and host populations. Solid grey and blue lines show the death and birth processes, respectively, in the tick and host populations. Solid green lines correspond to the transport of ticks by migratory birds or terrestrial hosts. Reproduction hosts are not competent hosts for transmitting the pathogen to ticks. See electronic supplementary material, appendix S2, for details on the Allee effect.

We built a reaction–advection–diffusion model based on a system of partial differential equations (PDEs) to simulate the northward invasion of ticks by migratory birds and terrestrial hosts across theoretical landscapes during three activity periods of migratory birds where ticks are active (northward migration, breeding and southward migration). A susceptible–infected epidemiological modelling framework was used to describe infection dynamics of a single pathogen in tick and host populations (figure 1). The theoretical landscapes of resource availability *G*(x) were generated from Gaussian random fields (GRFs), and each landscape consisted of a grid of 3000 × 5000 km with a cell resolution of 50 km (electronic supplementary material, appendix S1). All model parameters are listed in electronic supplementary material, appendix S3.

#### 2.1.1. Tick population dynamics

The model considered a population of ticks (*T*) including both immature and mature tick life stages. Although the tick population is not divided into distinct life stages (e.g. larvae, nymphs and adults), we explicitly consider the most important life-history traits: (i) new births in the tick population require adult ticks feeding on reproduction hosts and (ii) infection takes place when immature ticks feed on infected amplification or dilution hosts. The computational complexity (e.g. convergence of numerical solutions) of nonlinear reaction–advection–diffusion processes motivated this model simplification. The population of ticks was divided into classes of susceptible (i.e. uninfected; *S_T_*) and infected (*I_T_*) ticks such that the total density of ticks (i.e. number of ticks per km²) was *N_T_* = *S_T_* + *I_T_*. Tick population dynamics were given by the following equations:

 ∂ST(x,t)∂t=aT[−NT2+(KT++KT−+eT)NT+cT] NT⏟Birth −aT(eTNT+KT+KT−+cT)ST⏟Death −[αTAβA→T(IA/(NA+ND+NR))+αTDβD→T(ID/(NA+ND+NR))] ST⏟Infection +∇⋅(DT∇ST)⏟Random (diffusive) movementof ticks by hosts associatedwith a resource gradient−∇⋅(CTST)⏟Directed (advective) movementof ticks by hosts associatedwith a resource gradient

and

 ∂IT(x,t)∂t =[αTAβA→T(IA/(NA+ND+NR))+αTDβD→T(ID/(NA+ND+NR))]ST⏟Infection −aT(eTNT+KT+KT−+cT)IT⏟Death +∇⋅(DT∇IT)⏟Random (diffusive) movementof ticks by hosts associatedwith a resource gradient −∇⋅(CTIT)⏟Directed (advective) movementof ticks by hosts associatedwith a resource gradient.

Here, *S_T_*(x, *t*) and *I_T_*(x, *t*) represent the densities of susceptible and infected ticks, respectively, at spatial location x = (*x*, *y*) in the landscape at time *t*.

Tick populations can experience a strong mate-finding Allee effect [30], which can affect their establishment success in new areas. Ticks can be found in locations (on-host or off-host) where conspecific ticks are not necessarily present, which can result in a low tick population growth rate due to female and male ticks having difficulty finding one another to mate at low population density. Assuming a strong mate-finding Allee effect in our epidemiological model, we defined a density-dependent *per capita* birth function *b*_Allee_(*N_T_*) ≥ 0 and a density-dependent *per capita* mortality function *m*_Allee_(*N_T_*) ≥ 0 similarly to [31] as follows:

$$b_{\mathrm{Allee}}(N_{T})=a_{T}[-N_{T}^{2}+(K_{T+}+K_{T-}+e_{T})N_{T}+c_{T}]$$

and

$$m_{\mathrm{Allee}}(N_{T})=a_{T}(e_{T}N_{T}+K_{T+}K_{T-}+c_{T}),$$

where *K_T_*_+_ > 0 is the environmental carrying capacity for the tick population (e.g. density-dependent constraints on the availability of shelters for off-host and on-host ticks), *K_T_*_−_ with 0 < *K_T_*_−_ < *K_T_*_+_ represents the minimum viable population density below which a disease-free tick population is expected to go extinct and *a_T_* > 0 corresponds to the maximum *per capita* population growth rate. The parameters *e_T_* ≥ 0 and *c_T_* ≥ 0 determine the effects of density dependence and density independence in the demographic functions, respectively. More details on Allee effect calculations are given in electronic supplementary material, appendix S2. In our epidemiological model, all ticks are born susceptible to infection.

Tick infection occurs when susceptible immature ticks feed on an infected host. Assuming that pathogen transmission is frequency-dependent (i.e. it depends on the proportion of infected hosts [32]), the rate at which susceptible immature ticks become infected following a blood meal from an infected host (*λ_T_*) is defined as the product of the rate at which immature ticks (*n_j_N_T_*, where *n_j_* is the proportion of immature ticks) encounter competent hosts of type *C* (i.e. *C* = *A* for amplification hosts or *C* = *D* for dilution hosts; *α_TC_*), the probability that an infected host of type *C* transmits the pathogen to a susceptible immature tick (*β_C_*_→_*_T_*) and the proportion of infected hosts of type *C* $\left(I_{C}/(N_{A}+N_{D}+N_{R})\right)$. This gives the following equation:

$$\lambda_{T}=\alpha_{TA}\beta_{A\to T}\left(\frac{I_{A}}{N_{A}+N_{D}+N_{R}}\right)+\alpha_{TD}\beta_{D\to T}\left(\frac{I_{D}}{N_{A}+N_{D}+N_{R}}\right).$$

#### 2.1.2. Host population dynamics

The model considered a population of pathogen amplification hosts (i.e. hosts that can infect many ticks; *A*), a population of pathogen dilution hosts (i.e. hosts that infect ticks at lower rates than amplification hosts; *D*) and a population of tick reproduction hosts (*R*) that are incompetent for transmitting the pathogen to ticks. While the population of reproduction hosts consisted of a single class that defines the total density of reproduction hosts (*N_R_*), we separated the populations of amplification and dilution hosts into classes of susceptible (*S*) and infected (*I*) individuals so that the total density of individuals (i.e. number of individuals per km²) was *N* = *S* + *I*. Host population dynamics were given by the following equations:

Amplification host population:

$$\begin{array}{rl} \frac{\partial S_{A}(x,\;t)}{\partial t} & =\underset{\mathrm{Birth}}{\underbrace{b_{A}(1-(r_{A}/b_{A}K_{A})S_{A})N_{A}}}-\underset{\mathrm{Death}}{\underbrace{d_{A}S_{A}}}\\ & \;-\underset{\mathrm{Infection}}{\underbrace{\alpha_{TA}\beta_{T\to A}(I_{T}/(N_{A}+N_{D}+N_{R}))S_{A}}} \end{array}$$

and

$$\frac{\partial I_{A}(x,\;t)}{\partial t}=\underset{\mathrm{Infection}}{\underbrace{\alpha_{TA}\beta_{T\to A}(I_{T}/(N_{A}+N_{D}+N_{R}))S_{A}}}-\underset{\mathrm{Death}}{\underbrace{(d_{A}+(r_{A}/K_{A})N_{A})I_{A}}}.$$

Dilution host population:

$$\begin{array}{rl} \frac{\partial S_{D}(x,\;t)}{\partial t} & =\underset{\mathrm{Birth}}{\underbrace{b_{D}(1-(r_{D}/b_{D}K_{D})S_{D})N_{D}}}-\underset{\mathrm{Death}}{\underbrace{d_{D}S_{D}}}\\ & \;-\underset{\mathrm{Infection}}{\underbrace{\alpha_{TD}\beta_{T\to D}(I_{T}/(N_{A}+N_{D}+N_{R}))S_{D}}} \end{array}$$

and

$$\frac{\partial I_{D}(x,\;t)}{\partial t}=\underset{\mathrm{Infection}}{\underbrace{\alpha_{TD}\beta_{T\to D}(I_{T}/(N_{A}+N_{D}+N_{R}))S_{D}}}-\underset{\mathrm{Death}}{\underbrace{(d_{D}+(r_{D}/K_{D})N_{D})I_{D}}}.$$

Reproduction host population:

$$\frac{\partial N_{R}(x,t)}{\partial t}=\underset{\mathrm{Birth}}{\underbrace{r_{R}(1-N_{R}/K_{R})N_{R}}}.$$

While *S_A_*(x, *t*), *I_A_*(x, *t*), *S_D_*(x, *t*) and *I_D_*(x, *t*) represent the densities of susceptible amplification hosts, infected amplification hosts, susceptible dilution hosts and infected dilution hosts, respectively, at spatial location x = (*x*, *y*) in the landscape at time *t*, *N_R_*(x, *t*) defines the total density of reproduction hosts.

Similarly to other tick–host–pathogen dynamic models (e.g. [32]), we assumed that each host population of type *H* (i.e. *H* = *A* for amplification hosts, *H* = *D* for dilution hosts and *H* = *R* for reproduction hosts) experience density-dependent constraints due to intraspecific competition for resources and thus exhibit logistic growth with maximum *per capita* growth rate *r_H_* and environmental carrying capacity *K_H_*. We varied *K_H_* according to resource availability *G*(x) in the landscape using a linear relationship given by *K_H_*(x) = *G*(x)*K_H_*_0_, where *K_H_*_0_ is the maximum carrying capacity of hosts of type *H*. All hosts are born susceptible to infection at a *per capita* rate *b_H_* and die at a *per capita* rate *d_H_* whether they are infected or not.

Competent hosts of type *C* become infected when they are bitten by an infected immature tick at a rate *λ_C_*. This rate depends on the encounter rate between immature ticks and competent hosts of type *C* (*α_TC_*)*,* the probability that an infected immature tick transmits the pathogen to a susceptible competent host of type *C* (*β_T_*_→_*_C_*) and the ratio of ticks to hosts $\left(I_{T}/(N_{A}+N_{D}+N_{R})\right)$. This leads to the following equations:

$$\lambda_{A}=\alpha_{TA}\beta_{T\to A}\left(\frac{I_{T}}{N_{A}+N_{D}+N_{R}}\right)$$

and

$$\lambda_{D}=\alpha_{TD}\beta_{T\to D}\left(\frac{I_{T}}{N_{A}+N_{D}+N_{R}}\right).$$

#### 2.1.3. Tick movement by hosts

We considered the situation where resource availability induces variation in movement behaviour of hosts by changing their movement speed and turning frequency. The changes in movement patterns of hosts were modelled based on diffusion and advection rates. To achieve more realism in northward invasion patterns of ticks, we varied these rates according to three activity periods of migratory birds where ticks are active: bird migration to the north in spring, bird breeding during spring and summer and bird migration to the south in late summer and autumn. For the northward and southward migration periods, the diffusion and advection rates were calculated using movement parameters of migratory birds, whereas for the bird breeding season, the rates were defined according to movement parameters of terrestrial hosts. We thus expect that tick movement by hosts is more directed towards the north or south during the migration periods and more diffusive during the breeding season, which should lead to long-distance spread of ticks and the pathogen among resource-rich areas during the migration periods and short-distance spread within resource-rich areas during the breeding period. Among migratory passerine birds capable of transporting ticks and pathogens over long distances, American robins (*Turdus migratorius*) reduce their dispersal distance (mean: 142 m, range: 4–1200 m) due to nest protection and fidelity during the breeding season [33]. Thus, bird movement patterns are expected to be similar to those of terrestrial hosts during the breeding period. Each bird activity period lasted 90 days which gives a total tick activity period of 270 days over 1 year. The diffusion term *D_T_*(x, *t*) describes random movement of ticks by hosts in the landscape, while the advection term $C_{T}(x,\;t)$ defines directed movement of ticks by hosts towards the northern or southern part of the landscape. To represent the tendency for individuals to move slowly and spend more time in resource-rich areas, we assumed that the movement length was an exponentially decreasing function of resource availability *G*(x) [15]. The diffusion and advection terms were as follows:

$$D_{T}(x,\;t)=e^{-\omega_{G}G(x)}\eta_{T},$$

$C_{T}(x,\;t)=e^{-\omega_{G}G(x)}(\hat{v}/∥\hat{v}∥)\varepsilon_{T}$ with $\hat{v}=\psi_{T}x_{T}+(1-\psi_{T})(\nabla G(x)/$ ∥∇G(x)∥),

where *G*(x) represents the probability that spatial location x = (*x*, *y*) is in a rich-resource area (electronic supplementary material, appendix S1), the parameter *ω_G_* describes the sensitivity to rich-resource areas, the parameter *ψ_T_* controls the proportion of ticks moving northward and southward according to the bird activity period and ***x****_T_* is a unit vector directed from x towards the north or south. The parameters *η_T_* and *ɛ_T_* correspond to the diffusion and advection rates, respectively. We measured the magnitude of the direction vector $\hat{v}$ by its Euclidean norm (represented by ∥ ∥).

### 2.2. Model parameterization

The model was parameterized for North American Lyme disease from empirical and modelling studies, together with expert opinion on the bacterium *B. burgdorferi*, the black-legged tick (*I. scapularis*), the white-footed mouse (*Peromyscus leucopus*), the white-tailed deer (*Odocoileus virginianus*) and passerine birds including the American robin (*T. migratorius*), the ovenbird (*Seiurus aurocapilla*), the veery (*Catharus fuscescens*) and the wood thrush (*Hylocichla mustelina*). For literature-derived known input parameters, ranges of uncertainty were defined by setting the minimum and maximum as 30% lesser and greater than the default values. A calibration analysis (CA) was used to define a range of reasonable values for each model input parameter that was considered as highly uncertain or unknown. The CA was based on an approximate Bayesian computation analysis [34] (electronic supplementary material, appendix S4). The results of the CA are shown in electronic supplementary material, appendix S4 (table S1 and figure S1).

### 2.3. Model simulations

In North America, most tick-infested migrating passerine birds reside in the United States or further south during winter and move northward into Canada for the breeding season [27]. The populations of ticks in the United States appear to be in a state of flux driven by deer populations and changes in land use [35]. In our model, the tick movement by migratory birds was thus oriented along a north–south axis and tick flux occurred from southern latitudes. We imposed zero-flux boundary conditions except for the south of the theoretical landscape, meaning that ticks cannot enter or leave the study area. To the south, we applied Dirichlet boundary conditions where the tick flux can occur. Each simulation was initialized with a population of susceptible ticks occupying the southern 2% of the landscape. In each theoretical landscape represented by a two-dimensional rectangular mesh of 6000 bilinear quadrangular elements (60 in the *x* direction and 100 in the *y* direction), all nodes located in the first two element rows of the mesh were set to *K_T_*_+_. We introduced 13% infected ticks in the initial population of susceptible ticks [36]. At the model initialization time (i.e. *t* = 0), amplification, dilution and reproduction hosts were distributed across the resource gradient *G*(x) in the landscape with a number of individuals equal to *K_A_*, *K_D_* and *K_R_*, respectively. The simulation time-step was set to 1 day. The model ran during the tick activity period (270 days over 1 year) for 60 years until ticks reached the northern part of the landscape. In each theoretical landscape, the global spread rate of infected ticks (km/day), the tick infection prevalence (0–1) and the maximum density of infected ticks (number of ticks/km²) were calculated at each time-step of 10 days without significant loss of accuracy. The rate of global spread was calculated as the slope of the regression between the day of first detection of infected ticks at spatial locations (*x*, *y*) in the landscape and the distance between the infected locations and the nearest initial infected location (i.e. where the population of infected ticks was present at the model initialization [37]). A location was considered as infested by ticks if the population level of ticks at location (*x*, *y*) exceeded a certain threshold, reflecting local tick establishment. We tested four establishment thresholds that were calculated as 1%, 5%, 10% and 20% of tick carrying capacity. The system of PDEs was solved by a nonlinear finite-element method using the numerical simulation software MEF++ developed by the Groupe Interdisciplinaire de Recherche en Éléments Finis (GIREF; https://giref.ulaval.ca/) at the Université Laval, Québec (Canada). The time derivative was discretized by a two-step backward implicit scheme. The numerical method was second-order accurate in time and first-order accurate in space. The convergence tolerance of the Newton solver was set to 1 × 10^−1^ for all numerical simulations.

### 2.4. Sensitivity analysis

We performed a global sensitivity analysis (SA) to evaluate the sensitivity of predicted patterns of tick-borne pathogen spread to variations in model input parameters. A total of 30 input parameters were included in the SA due to the potential variability and uncertainty observed in real systems. We used Latin hypercube sampling to sample 1000 different combinations of the 30 parameters across a wide range of scenarios representing landscape–host–tick–pathogen interactions. As the theoretical landscapes were generated from GRFs that give rise to stochasticity in the values of resource availability *G*(x), we generated 10 replicated landscapes for each parameter combination, resulting in a total of 10 000 simulations. We used boosted regression tree (BRT) models [38] to explore the relative contribution of the 30 input parameters to model output variables, and thus to identify which of the input parameters had the greatest effect on these output variables (electronic supplementary material, appendix S5). We used the rate of infected tick spread, the tick infection prevalence and the maximum density of infected ticks as model output variables.

## 3. Results

Our BRT models performed well for quantifying the relative influence of ecological predictors on patterns of infected tick spread, tick infection prevalence and infected tick density with explained cross-validated deviance ranging from 88% to 99%, and with cross-validated correlation (correlation between the raw and predicted values of a given output variable) ranging from 0.94 to 0.99 (electronic supplementary material, table S1 in appendix S5). In the later sections, we describe the relationships between the model output variables and their four most influential input parameters (≥10% relative influence).

### 3.1. Rate of infected tick spread

**Figure 2.**
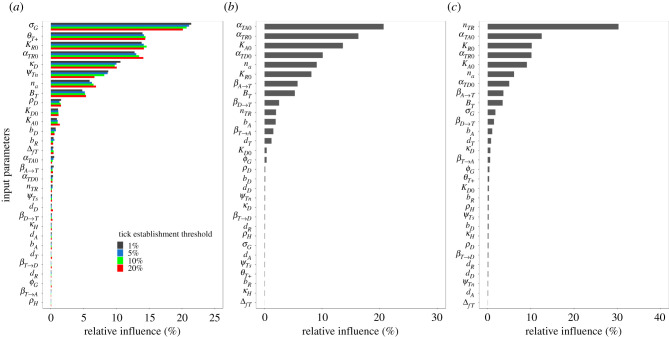
Relative influence of input parameters that are used to model the rate of infected tick spread (km/day) (*a*), tick infection prevalence (*b*) and maximum density of infected ticks (number of ticks/km²) (*c*).

**Figure 3.**
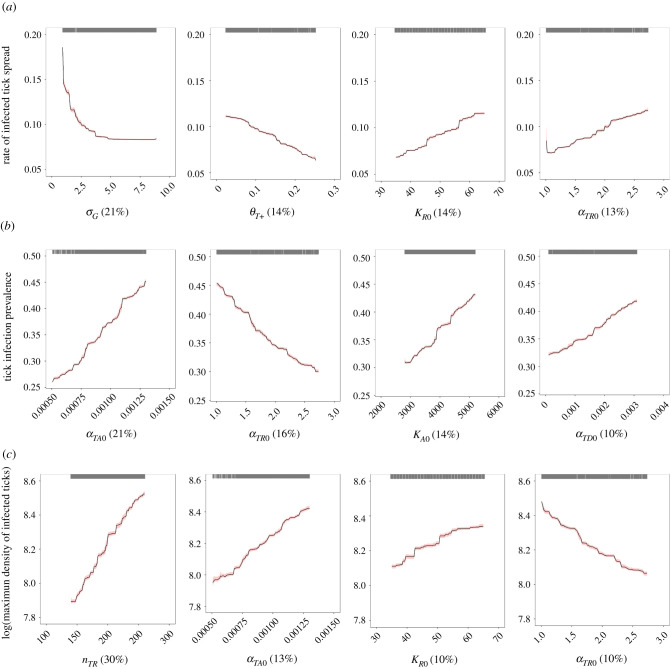
Partial dependency plots with bootstrapped 95% confidence intervals (red) for the four most influential input parameters predicting the rate of infected tick spread (km/day) with 5% tick establishment threshold (*a*), tick infection prevalence (*b*) and maximum density of infected ticks (number of ticks/km²) (*c*). The maximum density of infected ticks was log-transformed. Black tick marks at the top of plots represent raw data. Relative influence of each input parameter is in parentheses.

Rate of infected tick spread was primarily influenced by parameters associated with host movement (41%) and tick population dynamics (39%; electronic supplementary material, table S2 in appendix S5). Overall, there was no difference in the relative contribution of input parameters between the different tick establishment thresholds (figure 2 and electronic supplementary material, figure S1 in appendix S5). Four input parameters exerted a strong influence on the rate of infected tick spread (figure 2): the rate at which the daily distance moved by ticks on migratory birds and terrestrial hosts decreases with increasing resource availability (*σ_G_*; 21% relative influence), the Allee effect threshold (*θ_T_*_+_; 14% relative influence), the maximum carrying capacity of reproduction hosts (*K_R_*_0_; 14% relative influence) and the base rate at which adult ticks encounter reproduction hosts (*α_TR_*_0_; 13% relative influence). The base reproduction host-finding rate and the maximum carrying capacity of reproduction hosts displayed a positive relationship with the rate of infected tick spread (figure 3). Inversely, the rate of infected tick spread was negatively correlated with the Allee effect threshold and the parameter *σ_G_* that controls the sensitivity to resource-rich areas. The strongest pairwise interaction was weak (interaction size = 0.06) and not significant (*p* > 0.05), which suggests that the most influential predictors have additive rather than interacting effects on the rate of infected tick spread.

### 3.2. Tick infection prevalence

Tick infection prevalence was primarily influenced by parameters associated with the infection process (41%; electronic supplementary material, table S2 in appendix S5). The base rate at which immature ticks encounter amplification hosts (*α_TA_*_0_) had the largest impact on tick infection prevalence with a 21% relative influence (figure 2). The base reproduction host-finding rate (*α_TR_*_0_) was the next most influential parameter with a 16% relative influence. Tick infection prevalence was also sensitive to the maximum carrying capacity of amplification hosts (*K_A_*_0_; 14% relative influence) and the base rate at which immature ticks encounter dilution hosts (*α_TD_*_0_; 10% relative influence). The base amplification host-finding rate, the maximum carrying capacity of amplification hosts and the base dilution host-finding rate each showed a positive relationship with the tick infection prevalence (figure 3). Tick infection prevalence was also described by a negative relationship with the base reproduction host-finding rate. The strongest pairwise interaction was weak (interaction size = 0.09) and not significant (*p* > 0.05), indicating that the most influential input parameters have additive rather than interacting effects on tick infection prevalence.

### 3.3. Density of infected ticks

Density of infected ticks was primarily influenced by parameters associated with tick population dynamics (51%; electronic supplementary material, table S2 in appendix S5). The tick burden on reproduction hosts (*n_TR_*) was the most influential input parameter on the maximum density of infected ticks (30% relative influence) and was positively correlated with this output variable (figures 2 and 3). The latter was also described by a positive relationship with the base amplification host-finding rate (*α_TA_*_0_; 13% relative influence) and the maximum carrying capacity of reproduction hosts (*K_R_*_0_; 10% relative influence). The BRT models revealed a negative relationship between the maximum density of infected ticks and the base reproduction host-finding rate (*α_TR_*_0_; 10% relative influence; figure 3). The strongest pairwise interaction was weak (interaction size = 0.67) and not significant (*p* > 0.05), which suggests that the most influential predictors have additive rather than interacting effects on the maximum density of infected ticks.

## 4. Discussion

Many tick species have recently expanded their geographical range northward [39]. This ongoing invasion over large spatial scales presents a significant public and animal health threat. Fofana & Hurford [40] emphasize the need to develop epidemiological models integrating a mechanistic formulation of host movement to better understand the conditions for disease spread and occurrence. In this perspective, we built the first mechanistic movement model for tick-borne disease spread, parameterized for North American Lyme disease. Our findings highlight the importance of host movement behaviour and tick population dynamics in explaining the rate of infected tick spread into new regions, with tick infection prevalence and density of infected ticks driven by predictors related to the infection process and tick population dynamics, respectively. Identifying the role of different ecological processes driving tick-borne disease spread has wide-reaching applications and the modelling approach developed here could be easily applied to other tick–host–pathogen systems.

### 4.1. Rate of infected tick spread

Our results highlight the dual influences of movement behaviour of migratory birds and tick population dynamics on the speed of northward invasion of infected ticks. The attraction of migratory birds to resource-rich areas appears to play a substantial role in the pattern of northward invasion of ticks. Rapid spread rates are more likely to occur in areas that are unattractive to migrating birds. During their migration, birds spend much time at stopover sites (90% of their migration duration) where they can restore their energy stores and rest for subsequent migratory flights [41]. Since feeding ticks spend a fixed time attached to migrating birds before dropping off, longer stopovers are likely to reduce the total displacement distance of hitchhiking ticks moving northward. Changes in the stopover behaviour of migrating birds could, therefore, have important impacts on the spread of tick-borne diseases, as well as pathogen transmission. For example, agriculture intensification and habitat loss caused by human activities can reduce the number of stopover sites and thus constrain birds to using remaining sites [1], which could lead to either increased exposure to host-seeking ticks if the remaining sites are suitable for ticks or decreased exposure if the residual stopover sites are unsuitable for ticks.

In our simulation study, tick population dynamics also had a strong influence on infected tick invasion patterns. Specifically, we found that tick populations that experience a low threshold of mate-finding Allee effect are more likely to invade new areas quickly. The critical tick density below which the tick population dies out must be low (less than 10% of the tick carrying capacity) for the invasion to be successful. Our results provide evidence that Allee effects can shape tick invasion patterns and should be considered in epidemiological models of tick-borne pathogen spread. We also found that rates of infected tick spread were highest in areas with high encounter rates between adult ticks and reproduction hosts and large carrying capacities of reproduction hosts. Large-bodied reproduction hosts are an essential source of blood for adult female ticks that require a blood meal to produce viable eggs [22]. In North America, the white-tailed deer is a key reproduction host for adult ticks and contributes to the expansion and maintenance of tick populations in the environment [25]. Several empirical studies have reported a positive correlation between tick abundance and deer density (e.g. [42,43]).

### 4.2. Tick infection prevalence and density of infected ticks

Tick infection prevalence was mostly driven by input parameters related to infection dynamics, with the rates at which immature ticks encounter amplification and dilution hosts, and the carrying capacity of amplification hosts identified as key predictors. This underlines the importance of amplification hosts in the process of tick-borne disease transmission. Pathogen transmission is more likely to occur when amplification hosts are abundant in the environment [21], resulting in higher encounter rates with ticks. Changes in the ecology of amplification hosts should thus have important impacts on tick infection prevalence. In our study, birds were treated as dilution hosts that had a simple additive effect on the risk of contracting the pathogen, rather than a dilution effect that occurs when dilution hosts (i.e. poor-quality hosts for ticks and pathogens) divert tick blood meals away from amplification hosts [21,44], which is expected to reduce disease risk. In particular, the addition of dilution hosts increased the total carrying capacity of hosts in the landscape, which in turn increased the carrying capacity of ticks and thus led to amplification of the transmission cycle. The dilution hosts thus modulated pathogen dynamics in our tick–host–pathogen system.

The input parameters associated with tick population dynamics were important drivers of the density of infected ticks. Our results suggest that densities of infected ticks should be highest in areas with high encounter rates between immature ticks and amplification hosts, large carrying capacities of reproduction hosts and high levels of tick burdens on reproduction hosts. The tick burden on reproduction hosts controls the carrying capacity of ticks in our system of PDEs. This illustrates the critical role of reproduction hosts that drive the density of infected ticks (studies summarized in [32]). Thus, major changes in the ecology of reproduction hosts could have profound consequences on the abundance of infected ticks in the environment.

### 4.3. Implications for controlling tick-borne diseases

Kilpatrick *et al.* [45] emphasize the need to identify the key drivers of the tick-borne disease risk in order to more effectively target existing control strategies or develop new control methods. Our simulation study follows this line of research. Various methods have been explored to limit the density of infected ticks in the environment: reducing the density of amplification and reproduction hosts by hunting or predation [32,46], reduction of ticks on hosts or in the environment using acaricide treatments [47], modification of host species composition [48], vaccination of amplification hosts or humans [49], prevention of human exposure to tick bites through educational programmes [50] and landscape modification through habitat fragmentation [9], leaf litter removal [51] and controlled burning [52]. Our results suggest that control strategies that are effective at reducing tick burden on reproduction hosts should result in a reduced density of infected ticks in the environment. For example, the administration of acaricides to reproduction hosts has been shown to reduce adult tick burden on these hosts and, consequently, decrease the abundance of host-seeking immature ticks [47]. Several field studies assessed the effectiveness of the ‘4-poster’ passive topical treatment device that attracts deer to a corn bait source and applies an acaricide via rollers to the animal's neck, ears and head (studies summarized in [53]). Despite promising results, this method can be sensitive to the deployment of the 4-poster devices and local community of tick hosts [53].

It is also important to consider drivers of tick infection prevalence [45]. In our study, the rate at which immature ticks encounter amplification hosts was identified as the most influential predictor of tick infection prevalence. Consequently, control strategies that prevent ticks from coming into contact with or successfully feeding on amplification hosts are promising approaches for reducing the proportion of infected ticks in the environment. For example, the risk of contact between ticks and amplification hosts can be minimized through (i) management of amplification hosts by limiting their abundance which can include the protection of natural predators [54] or by encouraging species diversity in communities of immature tick hosts with a combination of competent and less competent reservoir species [48], (ii) management of ticks feeding on amplification hosts by topical or oral acaricides [47] or (iii) landscape management by creating more hostile environments for ticks in order to limit their survival and reproduction [55]. Functional connectivity (i.e. the adjustment of host movement to landscape attributes) and ecotones (i.e. zone of transition between two contrasting ecological systems) could also affect interactions between ticks and amplification hosts [56,57]. Nevertheless, we still lack a detailed understanding of how space use patterns of amplification hosts influence tick burden.

Our results highlight the importance of host movement behaviour in the process of tick-borne pathogen invasion, and specifically the key role of long-distance dispersal of migratory birds for understanding and predicting the spread of *I. scapularis* ticks and their associated pathogens. Studies of bird migration may therefore allow better prediction of future northward range expansion of tick species [26,58]. In many epidemiological models applied to tick-borne diseases (e.g. [59,60]), dispersal of migratory birds in heterogeneous landscapes is not explicitly considered because modelling movement behaviour often represents a technical challenge. Consequently, a simple migration rate is often used in these models when movement processes are not of primary interest in the study. Modelling approaches including mechanistic movement processes should improve the ability to anticipate spatial dynamics of ticks and their pathogens in heterogeneous landscapes. The mechanistic inclusion of host movement provides a better estimation of pathogen transmission events at the tick–host interface because movement processes govern contacts between ticks and hosts [40].

The model presented in this study describes infection dynamics of a single infectious agent within populations of hosts and ticks experiencing density-dependent constraints on population growth and incorporates host movement rules describing movement direction, velocity and turning frequency based on resource availability. Since our results show that long-distance dispersal of migratory birds is an important process in tick-borne pathogen invasion, our model is relevant for pathogens that spread by migratory birds, like *Borrelia* spp., *Rickettsia* spp., *Babesia* spp. and tick-borne encephalitis virus [61]. Although our calibration analysis ensures that the model reproduces realistic tick-borne pathogen spread patterns, we recommend that the model be validated in real landscapes by comparing simulated spatial abundance patterns of ticks with independent field data. While this study assesses the relative importance of ecological drivers on the spread of tick-borne pathogens from a theoretical perspective, our model can be easily applied to real landscapes worldwide in which the carrying capacities of hosts vary according to habitat suitability criteria. In the field of movement ecology, mechanistic movement models have been used to explore animal species population dynamics such as conspecific interactions, territoriality and landscape structure in home-range formation [10,11]. These population dynamic features could extend our model to new research perspectives on tick-borne disease ecology. Finally, the application of mechanistic movement models to scenarios of combined changes in climate and land use could significantly advance the understanding of tick-borne disease ecology and could contribute to important improvements in prevention and control strategies for these diseases.

## Data accessibility

Data are available from https://gitlab.com/otardy/ecological-drivers-of-tick-borne-pathogen-spread.
